# A Survey of Correlation Between Insulin-Like Growth Factor-I (IGF-I) Levels and Severity of Liver Cirrhosis

**DOI:** 10.5812/hepatmon.6181

**Published:** 2013-02-23

**Authors:** Asghar Khoshnood, Mohsen Nasiri Toosi, Mohammad Jafar Faravash, Alireza Esteghamati, Hosein Froutan, Hadi Ghofrani, Mohammad Kalani, Arash Miroliaee, Ahmad Abdollahi, Andrabi Yasir

**Affiliations:** 1 Department of Internal Medicine, Shahid Sadoughi Hospital, Shahid Sadoughi University of Medical Sciences, Yazd, IR Iran; 2 Department of Gastroenterology, Imam Khomeini Hospital, Tehran University of Medical Sciences, Tehran, IR Iran; 3 Department of Endocrinology, Imam Khomeini Hospital, Tehran University of Medical Sciences, Tehran, IR Iran; 4 Department of Pathology, Imam Khomeini Hospital, Tehran University of Medical Sciences, Tehran, IR Iran

**Keywords:** Insulin-Like Growth Factor I, Liver Cirrhosis, Child

## Abstract

**Background:**

Insulin-like growth factor is a polypeptide with endocrine, autocrine and paracrine effects which its structure is similar to the insulin molecule. While various tissues secrete IGF-1, 90% of the circulating IGF-1 is secreted by liver. Cirrhosis of liver is a condition accompanied by decreased level of IGF-1, in which the level of IGF-1 may be further decreased thorough the progression of the disease.

**Objectives:**

The aim of the present study was to demonstrate the relation between the IGF-1 levels and severity of liver disease according to Child- Pugh and Model for end stage liver diseases (MELD) Scores.

**Patients and Method:**

This was a descriptive-analytic cross sectional study performed on patients with cirrhosis admitted to gastroenterology clinic of Imam Khomeini Hospital in Tehran, Iran during the years 2007-2008. The diagnosis was based on liver biopsy. Initially for all patients, laboratory investigations including IGF-1, CBC, liver Enzymes, Alkaline phosphates, serum Albumin, Creatinine, direct and total Bilirubin were conducted. Also ultrasound and endoscopy were performed for evaluation of ascites and varices.

**Results:**

100 patients with cirrhosis with a male to Female ratio of 63:37 and a mean age of 44.4 ± 15 years were enrolled in the study. Median IGF-1 was 92.95 ± 91.51 ng/mL. 14 patients (14%) had IGF-1 within normal limits while 86 patients (86%) had abnormal IGF-1 levels. In all patients the correlation coefficient between IGF-1 and MELD was -0.317 (P = 0.001) and 0.478 between IGF-1 and Child- Pugh (P < 0.001).

**Conclusions:**

Our findings showed that IGF-1 can be used as an index for evaluating the severity of cirrhosis; also it can be used for determining the severity of the disease, when liver biopsy is not possible.

## 1. Background

Insulin like growth factor-1 (IGF-1) is a polypeptide with endocrine autocrine, paracrine effect which has some structural similarities with the insulin molecule (1). IGF-1 levels can be measured in the blood in 10-1000 ng/ml amounts. As levels do not fluctuate greatly throughout the day for an individual person, IGF-1 is used by physicians as a screening test for growth hormone deficiency and its excess in acromegaly and gigantism. Normal range values of IGF-1 have been determined in detail for specific age groups. Because of the wide range of hormone value, normal ranges have been reported by percentiles for each age group (2, 3). While various tissues secretes IGF-1, 90% of the circulating IGF-1 is secreted by liver (4). The release of IGF-1 from liver is stimulated by the growth hormone (5). Hepatocytes contain GH receptors; in which stimulation of these receptors increases the production and secretion of IGF-1 from the hepatocytes into plasma (6). IGF-1 is an anabolic hormone which causes a decrease in proteolysis and an increased stimulation of protein production, followed by an increase in muscular mass (7, 8). Liver cirrhosis is a state accompanied by a decrease in IGF-1 and progression of the disease (9-13). In liver cirrhosis IGF-1 level would be decreased while growth hormone would be increased (14). This decrease in IGF-1 is because of two factors; firstly, the decrease in growth hormone receptors in patients with cirrhosis and secondly progressive reduction in production of IGF-1 that is due to a decrease in hepatocytes (15-17). Increase in growth hormone is due to loss of inhibitory effect of IGF-1 on the hypothalamus or the hypophysis. Unresponsiveness of hepatocytes to growth hormone in child C will be determined by minimum rise of IGF-1 level (< 10%) in response to the growth hormone compared to more than 20% rise in normal population (9). Some nutritional and metabolic factors are effective on decreased levels of IGF-1. In fact, patients with cirrhosis have malnutrition like state, which is similar to patients undergoing prolonged fasting i.e. increased gluconeogenesis from liver and muscles (18-20). Child-Pugh scoring system is a suitable clinical method to determine the severity of disease in liver cirrhosis, and also involves qualitative criteria such as encephalopathy and ascites in addition to quantitative criteria, therefore can be used for staging (21) (Table 1). Child-Pugh class will be categorized in three classes, class A (a score of 5–6, one year's survival of 100% and two years survival of 85% ), Class B (7–9, one year's survival of 81% and two years survival of 57%), Class C (10 or above, one year's survival of 45% and two years survival of 35%). Similarly the Model for End stage liver disease (MELD) scoring system is a method in which qualitative criteria (Bilirubin, creatinine and INR) used to determine the severity of liver involvement. Score will be calculated by the following formula (22): MELD = 0.957 × loge [creatinine (mg/dL)] + 0.378 × loge [Bil (mg/dL)] + 1.12 × loge (INR) + 0.643. In interpreting the MELD Score in hospitalized patients, the 3 month mortality is (23):

40 or more — 71.3% mortality

30–39 — 52.6% mortality

20–29 — 19.6% mortality

10–19 — 6.0% mortality

< 9 — 1.9% mortality

**Table 1:** Child-Pugh Classification of Cirrhosisa

Grade Parameter	Classification of Cirrhosis		
	A	B	C
**Albumin**	> 3.5 g/dL	3-3.5 g/dL	< 3 g/dL
**Bilirubin**	< 2 mg/dL	2-3 mg/dL	> 3 mg/dL
**Protrombin** **time**	< 15 sec	15-17 sec	> 17 sec
**Ascites**	without ascites	mild to moderate ascites	severe ascites
**Encephalopathy**	without encephalopathy	mild encephalopathy	advanced encephalopathy

^a^Child-Pugh class A (a score of 5–6, one year's survival of 100% and two years survival of 85% ), Class B (7–9, one year's survival of 81% and two years survival of 57%), Class C (10 or above, one year's survival of 45% and two years survival of 35%)

## 2. Objectives

There are few studies on the role of IGF 1 as an index for determining the severity of the disease in patients with cirrhosis. Also detecting the relationship among IGF 1 and MELD and child-pugh criteria's is missed in existing studies. Therefore in this study, we decided to find the relation between IGF-1 and severity of liver disease according to the MELD and child-pugh criteria.

## 3. Patients and Methods

This descriptive-analytic cross sectional study was performed on patients with cirrhosis which were admitted to the gastroenterology clinic at Imam Khomeini Hospital, Tehran, Iran, during the years 2007-2008. The diagnosis was based on liver biopsy or clinical criteria. Exclusion criteria included advanced encephalopathy, Hepatorenal syndrome, recent infections, diabetes, chronic renal diseases, gastro-intestinal bleeding and past history of malignancy. In view of the study conducted by Vyzartiadis (24) where IGF-1 mean levels was calculated as 28.9 ± 3 in patients with cirrhosis, a sample size of at least 82 patients with an accuracy of 6.5% and a confidence limit of 95% was needed for our study. Initially the lab studies including AST, ALT, Alkaline phosphatase, Platelets, C.B.C, serum creatinine, serum albumin and Direct and total bilirubin were measured for all patients. Similarly ultrasound for the detection of ascites and endoscopy for varices were performed. Then severity of liver disease was determined according to Child-Pugh criteria (Table 1) and MELD score. The MELD score was calculated by the free software available on Mayo clinic website (http/www.mayoclinic.org/gi-rst/mayomode/5.html), which was based on the formula previously mentioned in introduction. Thereafter, IGF-1 measurement was performed using bio source kits, radioimmunoassay and protein binding methods. For this purpose specific amount of labeled IGF-1 and Iodine 125 (I 125) with the sample for which IGF-1 level had to be determined were placed in polyester test tubes. After two hours of centrifugation at room temperature and incubatory condition, the sediment was collected, and then IGF-1 concentration was determined by negative calibration. All the collected data was analyzed using SPSS-16. P value below 0.05 was considered as significant.

## 4. Results

**Figure 1:**
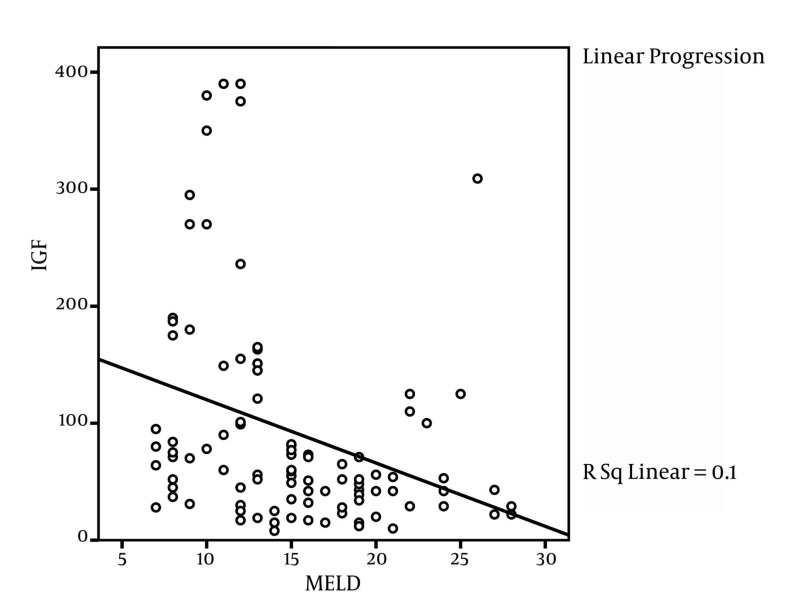
Linear Correlation Between MELD Score and IGF-1

**Figure 2:**
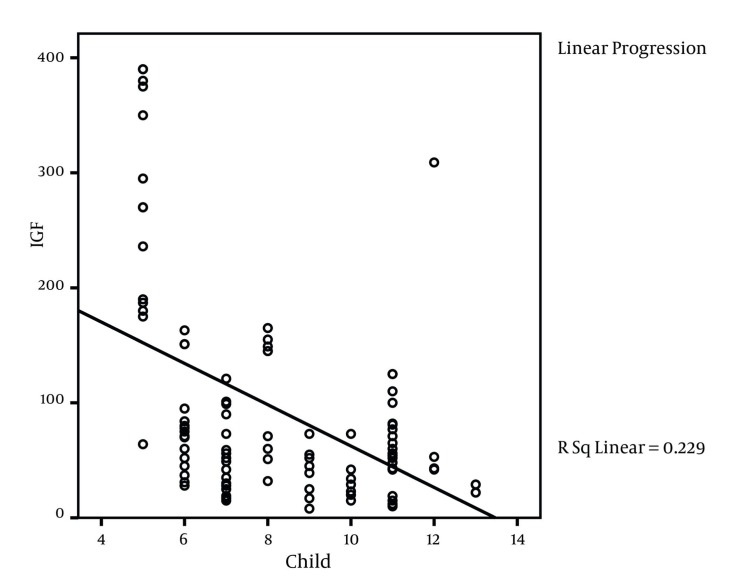
Correlation Between IGF-1 and Child-Pugh Score

This study was conducted on 100 liver cirrhosis patients. 63 patients (63%) were male and 37 (37%) were female. The mean age of patients was 44.4 ± 15 years with the range of 12 to74 years. Table 2 shows the frequency of etiologic factors of liver cirrhosis. Based on clinical information which was registered from patients` documents, 66 patients (66%) had esophageal varices, 82 (82%) had ascites (66 patients (66%) moderate ascites and 16 patients (16%) severe ascites) and 87 patients (87%) had splenomegaly. Mean IGF level was 92.95± 91.51. IGF-1 level in 14 patients (14%) was within the normal range while 86 patients (86%) had abnormal IGF-1 values. Table 3 indicates the ratio and the frequency of Child-Pugh classes according to the IGF state. The mean values for IGF-1 was 167.43 ± 121 ng/mL for class A, 64.65 ±45.13 for class B and 57.61 ± 52.9 for class C patients where the difference was significant among the groups (P < 0.001, ANOVA). Our study findings showed that MELD index mean value was 15 ± 5.3 in patients and correlation coefficient was -0.317 (P = 0.001, ANOVA) between IGF-1 and MELD index (Figure 1). Also correlation coefficient between IGF-1 and Child-Pugh score was -0.478 (P < 0.001) (Figure 2). Table 4 shows correlation coefficient among Child-Pugh score, MELD and IGF-1 according to sex. For further assessment of relation among IGF-1 and Child-Pugh and MELD indices, mean of MELD and Child-Pugh indices were calculated according to normal and abnormal values of IGF-1. Mean Child-Pugh score in patients with normal IGF-1 value was 5.5 ± 5.2 compared to 8.7 ± 2.2 in patients with abnormal IGF-1 values (P**< 0.001). Similarly mean MELD score in patients who had normal and abnormal IGF-1 levels was 11 ± 4.5 and 15.7 ± 5.2 respectively, which shows a significant difference (P = 0.002). Also correlation coefficient between IGF and age was determined as 0.445 (P < 0.001). There were no significant relation between gender and abnormal IGF-1 since 35.7% (five patients) female and 64.3% (nine patients) male had normal IGF-1 values while 27.3% (32 patients) of female and 62.8% (54 patients) of male patients had abnormal IGF-1 levels (P = 0.0914). Also there were no statistically significant difference between esophageal varices and IGF-1, as 64.3% of patients with normal IGF-1 values had esophageal varices compared to 66.3% of patients who had abnormal IGF-1 (P = 0.884) values. However, significant relation between IGF-1 level and splenomegaly was found since 35.7% of patients with normal IGF-1 values had splenomegaly compared to 95.3% of patients with abnormal IGF-1 (P < 0.001) levels. Table 5 shows correlation between IGF-1 and lab indices.

**Table 2:** Frequency of Etiologic Factors in the Development of Cirrhosis in PatientsUnder Study

Frequency Etiology	Patients, No. (%)
**Hepatitis B**	25 (25)
**Autoimmune hepatitis **	25 (25)
**Hepatitis C**	22 (22)
**Cryptogenic**	17 (17)
**Primary Sclerosing Cholangitis**	5 (5)
**Budd-Chiari Syndrome**	4 (4)
**Wilson**	2 (2)
**Total**	100

**Table 3:** Ratio and Frequency of Child-Pugh Classes According to the IGF-1 Level

IGF-1 Child Class	Normal IGF-1 value, No. (%) a	Abnormal IGF-1 value, No. (%) a	Total, No. (%)
**A**	13 (13)	17 (17)	30 (100)
**B**	0 (0)	34 (34)	34 (100)
**C**	1 (1)	36 (36)	36 (100)
**Total**	14 (14)	86 (86)	100 (100)

Abbreviation: IGF-1, insulin-like growth factor-1

^a^Normal:(90-360)

**Table 4:** Correlation CoefficientBetween Study Indices According to Gender (ANOVA)

Index	Correlation Co-Efficient	P value
**Male (n = 63)**		
IGF-1 - Child-Pugh score	0.412	0.001
IGF-1 - MELD	0.307	0.014
**Female (n = 37)**		
IGF-1 - Child-Pugh score	0.622	0.001
IGF-1 - MELD	0.4157	0.011

Abbreviation: IGF-1, insulin-like growth factor-1

**Table 5:** CorrelationBetween IGF-1 and Study Lab Indices (ANOVA)

Indices	Correlation Co-Efficient	P value
**IGF-1-bilirubin**	-0.188	0.061
**IGF-1-INR**	-0.261	0.009
**IGF-1-platelets**	0.361	0.001
**IGF-1-creatinine**	-0.278	0.005
**IGF-1-albumin**	0.645	0.001

Abbreviation: IGF-1, insulin-like growth factor-1

## 5. Discussion 

In our study, most of the patients were in the fourth and fifth decades of their lives and the mean age of patients was 44 years. The most common causes of liver cirrhosis in these patients were autoimmune hepatitis, Hepatitis B and Hepatitis C. These findings can suggest an increased prevalence of aforementioned diseases in the population or an increase in tendency of these diseases to progress towards liver cirrhosis. The relevant findings along with the laboratory investigations of patients is suspicious for pathologic changes in liver function as is expected in liver cirrhosis in a manner that blood bilirubin would be increased, while serum albumin and platelet count would be decreased. Serum creatinine in these patients was within the normal range after exclusion of patients with renal failure or Hepatorenal Syndrome. So far as staging of severity of liver disease was considered, our findings showed a uniform distribution of patients in Class A, B and C of Child-Pugh scoring system, however, with respect to MELD staging system, distribution of patients was towards the higher scores mostly. Our findings show that most of patients (nearly 85%) had abnormal IGF-1 level. Complications of cirrhosis had developed in most of our patients; we detected varices in 66%, ascites in 82% and splenomegaly in 87% of patients. In all patients a statistically significant relation among IGF-1 and MELD or IGF-1 and Child-Pugh score was found. An inverse relationship was present in both conditions and showed a significant relation with the severity of disease, which MELD or Child-Pugh scores were increased and IGF-1 was decreased. This relation was observed separately in both men and women and did not seem to have any relation with sex of the patients, however between age of patients and IGF-1 a significant inverse relationship was apparent. With an increase in age of patients with cirrhosis, a decrease in IGF-1 levels was observed. In a similar study performed in year 2004, 44 patients with advanced viral cirrhosis were divided into three groups according to Child's scoring and compared with 35 healthy controls. IGF-1 significantly decreased with the progression of cirrhosis and IGF-1 less than 30 ng/ml was associated with a poor prognosis (25). After distinguishing age of patients in our study, in spite of the fact that an acceptable correlation coefficient was present among IGF-1 and MELD and Child-Pugh score; however lacks of statistical significance on many occasions was detected. It can be owing to the low number of patients in each group. As the correlation coefficient is greatly dependent on the sample size, correlations that were not statistically significant should be interpreted cautiously. It is possible that after increasing the sample size in each age group, relation among these indicators in certain age groups would become significant. However, it seems that IGF-1 along with Child-Pugh or MELD indices are more significant in age groups (20-60 years) relative to younger age groups. More studies seem to be necessary in this regard. Our study shows that there is no significant relation between IGF-1 and varices which can be due to presence of other concordant conditions leading to varices than liver dysfunction. However, a meaningful relation was found among IGF-1 and ascites and splenomegaly; patients with splenomegaly and ascites formed a greater proportion of patients with abnormal results of IGF-1 test. In a similar study in 2003, 40 patients with advanced viral and alcoholic cirrhosis and primary biliary cirrhosis with variable severity according to Child staging were evaluated in which the level of IGF-1 was inversely related to the degree of splenomegaly and the severity of cirrhosis (24). Similarly, lab indices including albumin, INR, platelets, and to some extent bilirubin showed significant relation with IGF-1, also patients with abnormal results of IGF-1 test had these lab indices within the abnormal range. In a similar study performed in china in 2001, IGF-1 level was evaluated in patients with chronic liver disease and cirrhosis; lowest IGF-1 level was observed in patients with cirrhosis and also showed a relation with serum albumin level, to the extent that a severe decrease in IGF-1 level was seen at albumin level of < 3g/dL (26). The results of our study suggest that IGF-1 can be an index of severity of cirrhosis and also a marker of liver function; thus can be used for determining severity of the disease in patients in which liver biopsy is not possible.
